# The topology of connections between rat prefrontal and temporal cortices

**DOI:** 10.3389/fnsys.2015.00080

**Published:** 2015-05-20

**Authors:** Stacey A. Bedwell, E. Ellen Billett, Jonathan J. Crofts, Danielle M. MacDonald, Chris J. Tinsley

**Affiliations:** Division of Biosciences, School of Science and Technology, Nottingham Trent UniversityNottingham, UK

**Keywords:** prefrontal cortex, ordering, connections, temporal cortex, rat

## Abstract

Understanding the structural organization of the prefrontal cortex (PFC) is an important step toward determining its functional organization. Here we investigated the organization of PFC using different neuronal tracers. We injected retrograde (Fluoro-Gold, 100 nl) and anterograde [Biotinylated dextran amine (BDA) or Fluoro-Ruby, 100 nl] tracers into sites within PFC subdivisions (prelimbic, ventral orbital, ventrolateral orbital, dorsolateral orbital) along a coronal axis within PFC. At each injection site one injection was made of the anterograde tracer and one injection was made of the retrograde tracer. The projection locations of retrogradely labeled neurons and anterogradely labeled axon terminals were then analyzed in the temporal cortex: area Te, entorhinal and perirhinal cortex. We found evidence for an ordering of both the anterograde (anterior-posterior, dorsal-ventral, and medial-lateral axes: *p* < 0.001) and retrograde (anterior-posterior, dorsal-ventral, and medial-lateral axes: *p* < 0.001) connections of PFC. We observed that anterograde and retrograde labeling in ipsilateral temporal cortex (i.e., PFC inputs and outputs) often occurred reciprocally (i.e., the same brain region, such as area 35d in perirhinal cortex, contained anterograde and retrograde labeling). However, often the same specific columnar temporal cortex regions contained only either labeling of retrograde or anterograde tracer, indicating that PFC inputs and outputs are frequently non-matched.

## Introduction

Prefrontal cortex (PFC) is known to have a role in cognitive (Kolb, 1984; Fuster, 2001; Schoenbaum and Roesch, 2005), executive (Alvarez and Emory, 2006), emotional (Frysztak and Neafsey, 1991), and autonomic functions (Neafsey, 1990; Frysztak and Neafsey, 1991). Abnormalities of cortical connections have been linked to neurological/psychiatric disorders such as autism and schizophrenia (Kleinhans et al., 2008; Fornito and Bullmore, 2015). Anatomically, rat PFC includes the medial PFC, orbital frontal cortex and the agranular insular cortex. Rat medial PFC includes the prelimbic, infralimbic medial agranular, and anterior cingulate regions (Vertes, 2004, 2006). Orbital PFC regions include the medial orbital (MO), ventral orbital (VO), ventrolateral orbital (VLO), and lateral orbital (LO) areas (Krettek and Price, 1977; van de Werd and Uylings, 2008). Adjacent to these regions and on the lateral edge of the PFC lies the dorsolateral orbital area (DLO) and the agranular insular area (van de Werd and Uylings, 2008). Functionally, medial PFC (mPFC) has strong links to motor cortex and mPFC is thought to be involved in temporal processing (Narayanan and Laubach, 2006, 2008, 2009; Smith et al., 2010; Kim et al., 2013). Orbital PFC has a role in the prediction of future outcomes and is proposed to have an analogous functional role in rodents and primates (Schoenbaum and Roesch, 2005; Schoenbaum and Esber, 2010). Functionally, the agranular insular cortex and DLO have connections to neighboring gustatory insular cortex and have a putative sensory role (Gallagher et al., 1999; Fujita et al., 2011). Unlike other regions of the cerebral cortex there is currently little evidence for functional mapping of responses in rodent PFC, however there are topological distinctions to the functional characteristics of PFC regions (Cassaday et al., 2014).

PFC is known to have substantial connections to temporal cortex (Delatour and Witter, 2002; Hoover and Vertes, 2007, 2011; Agster and Burwell, 2009; Kondo and Witter, 2014). Studies have shown that there are projections from PFC to area Te, perirhinal cortex, and entorhinal cortex. In addition there is evidence that there are substantial projections from these temporal cortex regions back to PFC sites (Agster and Burwell, 2009).

There is evidence for topographical ordering in the projections from PFC to temporal cortex (Kondo and Witter, 2014), the projections from PFC to the striatum (Sesack et al., 1989; Berendse et al., 1992; Schilman et al., 2008), the projections from PFC to the epithalamus (Kim and Lee, 2012) and for topological ordering in the projections from PFC to motor cortex (Bedwell et al., 2014b). In addition at least two studies describe lamina-specific projections to and from the rat PFC. Lamina-specific projections are reported in the projection from sensory-motor cortex to different subdivisions of rat PFC (Bedwell et al., 2014b) and in the projections from mPFC to striatum in the rat (Gabbott et al., 2005).

Although, there is substantial evidence for the ordering of PFC connections, there is conflicting evidence concerning the fine-scale reciprocity of these connections. Reciprocal connections are thought to occur widely throughout the cerebral cortex and there is evidence for their occurrence in rat PFC (Cinelli et al., 1987; Datiche and Cattarelli, 1996; Little et al., 2009), temporal (Vaudano et al., 1991; Pitkanen et al., 2000; Kealy and Commins, 2011), somatosensory (Aronoff et al., 2010; Lee et al., 2011), and visual cortices (Olavarria and Montero, 1981; Miller and Vogt, 1984). Previous studies have reported evidence for both reciprocal (Ishikawa and Nakamura, 2003) and non-reciprocal (Agster and Burwell, 2009) connections of both PFC and temporal cortex. Reciprocal connections have been reported in the connections between perirhinal and pyriform cortex (Agster and Burwell, 2009). Additionally, the connections between lateral entorhinal cortex and secondary motor cortex and between postrhinal cortex and anterior cingulate cortex appear to be non-reciprocal (Agster and Burwell, 2009; Kealy and Commins, 2011). In the connections between sensory-motor cortex and PFC, reciprocal connections are recorded between mPFC and motor cortex however the somatosensory cortex provides an input to PFC that is not reciprocated in many columnar regions of cortex (Bedwell et al., 2014b). One of the problems with interpreting these studies is that most have not sought to address the question of fine-scale reciprocity.

**Figure 1:**
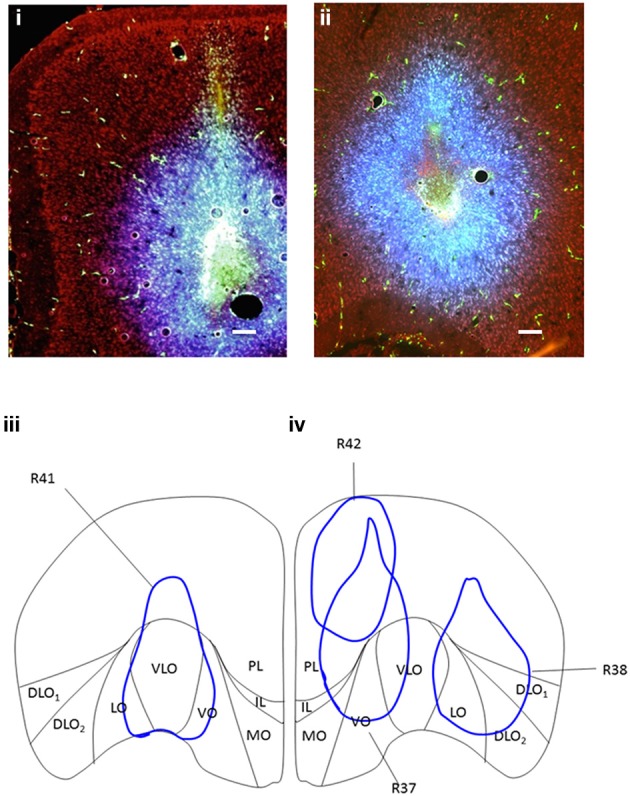
**(i)** Coronal section of PFC showing site of dual-injection of Fluorescein BDA (green) and Fluoro-Gold (blue) into VO (case R37). **(ii)** Coronal section of PFC showing site of dual-injection of Fluorescein BDA (green) and Fluoro-Gold (blue) into DLO (case R38). Propidium Iodide labeling is shown in red. **(iii)** Coronal cross section of PFC showing location and spread of (100 nl) BDA (fluorescein) and (100 nl) Fluoro-Gold dual-injection into VLO (R41-injection “C”) in the right hemisphere. **(iv)** Coronal cross section of PFC showing location and spread of (100 nl) BDA (fluorescein) and (100 nl) Fluoro-Gold dual-injections; Premlimbic (R42-injection “A”), Ventral Orbital (R37-injection “B”) and Dorsal Lateral Orbital (R38-injection “D”) in the left hemisphere. There is some overlap between the spread from injection sites (R42 and R37, R37 and R41, R38 and R41). Due to the use of combined injections of Fluoro-Gold and BDA, the spread from injections was greater than that produced from a single 100 nl injection. However, these injection volumes were chosen to ensure that significant labeling occurred in the target projection sites (for retrograde and anterograde label). Scale bars = 200 μm.

Here we sought to investigate the fine-scale reciprocity of PFC connections through an analysis of the ordering of input and output connections. We performed dual injections of anterograde and retrograde tracer into identical sites within the rat PFC and investigated the location of the associated connected sites (see Figure 1). A preliminary version of this report was previously published in abstract form (Bedwell et al., 2014a).

## Materials and methods

### Animals

#### Ethical statement

All Animal procedures were carried out in accordance with the UK Animals Scientific Procedures Act (1986), EU directive 2010/63 and were approved by the Nottingham Trent University Animal Welfare and Ethical Review Body.

#### Experimental animals

Twenty four male CD rats were obtained from Charles River, UK. On receipt the animals were examined for signs of ill-health or injury. The animals were acclimatized for 10 days during which time their health status was assessed. At the start of the surgery the animals weight range was 294–371 g.

Prior to surgery the animals were housed together in Individually Ventilated Cages (IVC) (Techniplast double decker Greenline rat cages). The animals were allowed free access to food and water. Mains drinking water was supplied from polycarbonate bottles attached to the cage. The diet and drinking water were considered not to contain any contaminant at a level that might have affected the purpose or integrity of the study. Bedding was supplied by IPS Product Supplies Ltd in form of 8/10 corncob. Environmental enrichment was provided in the form of wooden chew blocks and cardboard fun tunnels (Datesand Ltd., Cheshire, UK). Post-surgery the animals were individually housed in the same conditions. The animals were housed in a single air-conditioned room within a barrier unit. The rate of air exchange was at least 15 air changes per hour and the low intensity fluorescent lighting was controlled to give 12 h continuous light and 12 h darkness. The temperature and relative humidity controls were set to achieve target values of 21 ± 2°C and 55 ± 15%, respectively.

#### *In vivo* observations

Individual bodyweights were recorded on Day-10 (prior to the start of dosing) and daily thereafter. All animals were examined for overt signs of ill-health or behavioral change immediately prior to surgery dosing, during surgery and the period following surgery. There were no observed clinical signs/symptoms of toxicity or infection. There was no significant effect on body weight development detected.

#### Surgical and experimental procedures

Rats were anesthetized with isoflurane (Merial, Harlow, UK) and placed in a stereotaxic frame with the incisor bar set so as to achieve a flat skull. Buprenorphine (0.05 mg/kg i.m/s.c) and Meloxicam (up to 1 mg/kg s.c/orally) analgesia were provided peri-operatively and for several days post-operatively. Body temperature was monitored during and immediately after surgery using a rectal thermometer. Craniotomies (<1 mm diameter) were made at predetermined stereotaxic coordinates. Sterile tracer solution was deposited into the PFC cortex by injection, via a 0.5 μl Neuro-syringe (Hamilton, Germany) and delivery was controlled via use of a manual microsyringe injection holder (World Precision Instruments, catalog# 502245). Dual-injections of anterograde (2.5% (w/v) Fluorescein biotinylated dextran amine (BDA); catalog #: SP-1130 or 10% FluoroRuby in distilled water, Fluorochrome, Denver, Colorado) and retrograde tracer (4% (w/v) Fluoro-Gold in distilled water, Fluorochrome) were made into the prelimbic (PL), VO, VLO, or dorsolateral orbital cortex (DLO), with the intention of revealing the anatomical connections of PFC regions (this was performed via a first injection of Fluoro-Gold followed by a second injection of BDA into the same injection site). The distance between craniotomy co-ordinates (usually 1 mm) was based on the measured spread of tracers in preliminary and the present studies (~1 mm in diameter). The tracer injections were all made with the injection needle orientated vertically. Injections were made at AP 3.7 mm from Bregma (A) ML 1.2, 2.4 mm below cortical surface, (B) ML 1.2, 3.2 mm below cortical surface, (C) ML 2.2, 3.2 mm below cortical surface (D) ML 3.2, 3.2 mm below cortical surface and targeted with the use of a stereotaxic atlas (Paxinos and Watson, 1998).

Each rat received dual-injections of retrograde tracer and anterograde tracer into the same injection site. Rats received injections of (1) Fluoro-Gold (100 nl) at a rate of 100 nl/min and (2) BDA-Fluorescein (100 nl) at a rate of 100 nl/min or Fluoro-Ruby at a rate of 10 nl/min into various subdivisions of PFC, separated by 1 mm (Figure 1; Separate labeling of the Fluoro-Gold and BDA-Fluorescein at these injection sites is shown Figure S1 of the Supplementary Material). Three of the dual-injections of Fluoro-Gold and BDA-Fluorescein were made into the left hemisphere of separate rats (rats R37, R38, and R42) and one dual-injection was made in the right hemisphere in another rat (R41). The dual-injections of Fluoro-Gold and Fluoro-Ruby were made into the left hemisphere of separate rats (rats R39, R40, R43, and R44; Sites for these injections are shown in Figure S2 of the Supplementary Material). Additional separate injections of Fluoro-Gold (left hemisphere: rat R15; right hemisphere: rats R4, R5, R6, R7), BDA-Fluorescein (left hemisphere: rat R7; right hemisphere: rats R1, R3, R8) and Fluoro-Ruby (right hemisphere: rats R9, R19, R20, R29) were also made. Eight additional and equivalent Fluoro-Gold injections were made into the left (animal labels: R9, R12, R13, R16, R19, R20, R21) and right (R5) hemisphere of different animals to verify whether the location and ordering of projections differed on the either side of the brain (the results of this analysis are not included in the Results Section). The same overall order and positioning of Fluoro-Gold labeling was observed on both sides of the brain. Some of these animals (R1, R3, R4, R5, R6, R7, R8, R9, R13, R16, R19) were used in a previous study that investigated the connections between the same PFC injection sites and sensory-motor cortex (Bedwell et al., 2014b). The size of spread from injections was very consistent between injections of the same volume and tracer, the diameter of spread resulting from dual injections was larger than that produced from single injections (dual injections were 26% larger).

Following a survival time of 7–9 days, the rats were deeply anesthetised with pentobarbital (Sigma-Aldrich, UK), and transcardially perfused with 0.1 M phosphate buffered saline (PBS, pH7.4) (~200 ml) followed by 4% paraformaldehyde (PFA) (~200 ml) in PBS, pH7.4. The brain was subsequently removed and stored for 24 h in 4% PFA in 0.1 M PBS, followed by cryoprotection in 30% sucrose in 0.1 M PBS.

### Anatomical processing and microscopy

#### Anatomical processing

For analysis of connections, two series of 40 μm coronal sections were taken (2 in 6 sections) on a freezing microtome (CM 1900, Leica, Germany). Sections were mounted onto gelatin coated slides. One series was processed by implementing the avidin-biotin method (Vectastain® ABC, Vector Laboratories, CA), for bright field imaging of Fluorescein labeled axon terminals. This series of sections was counterstained with thionin. For analysis of Fluoro-Gold labeled cells and the fluorescently labeled injection sites, a parallel series of 40 μm coronal sections was taken, mounted onto gelatin coated slides, then cover slipped with Vectashield® mounting medium (with propidium iodide) for fluorescent imaging of Fluoro-Gold (for the injection site and labeled cells), Vectashield with 4′,6-Diamidino-2-phenylindole (DAPI) for fluorescent imaging of the Fluoro-Ruby injection site and labeled axon terminals and Fluorescein BDA (for the injection site).

**Figure 2:**
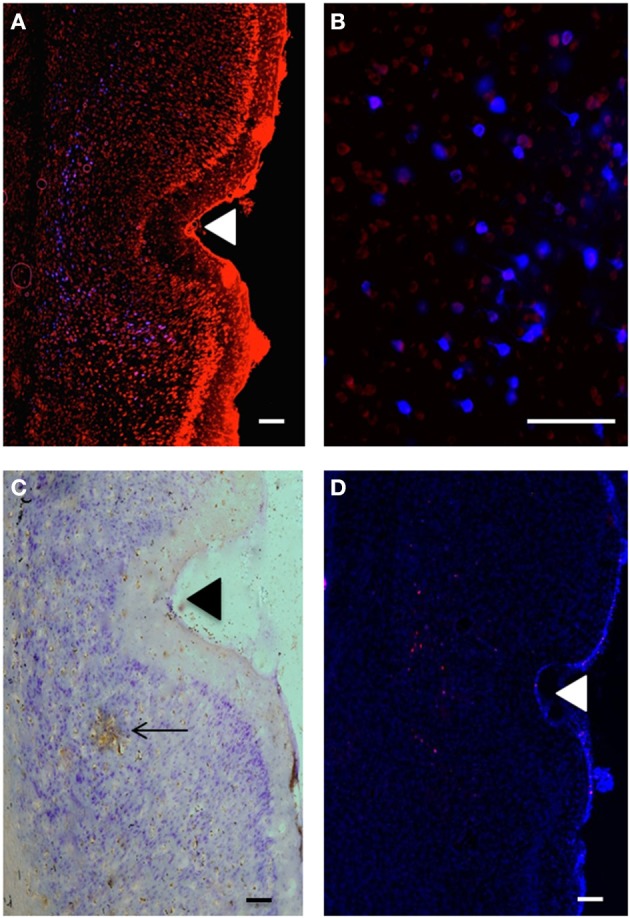
**(A)** Retrogradely labeled cells (blue) in temporal cortex at 3.1 mm posterior to bregma; perirhinal areas 35d, 36v, 36d and area Te, produced by injection of Fluoro-Gold (100 nl) into VO (injection B: R37). Propidium Iodide labeling is shown in red. Scale bar = 200 μm. **(B)** Retrogradely labeled cells (blue) in temporal cortex; perirhinal cortex (3.6 mm posterior to bregma), produced by injection of Fluoro-Gold (100 nl) in VO (injection B: R37). Propidium Iodide labeling is shown in red. Scale bar = 100 μm. **(C)** Anterogradely labeled area (brown) in perirhinal cortex (area 35d), produced by injection of BDA (100 nl) into VLO (injection C: R41). Scale bar = 200 μm. Arrow shows area of intense DAB staining of axon terminals. Other brown staining indicates less intense anterograde labeling. **(D)** Anterograde labeling (red) in temporal cortex produced by injection of Fluoro-Ruby (100 nl) into PL (injection A:R44). DAPI labeling is shown in blue. Triangles indicate the location of the rhinal sulcus.

Sections were examined using either bright field (Fluorescein) or fluorescent microscopy (Fluorescein, Fluoro-Gold, and Fluoro-Ruby). Fluorescent photos were captured of the injection sites and retrogradely labeled cells (Fluoro-Gold, see Figures 2A,B) and axon terminals labeled by Fluoro-Ruby (see Figure 2D). Brightfield photos were captured of anterogradely DAB labeled areas (Fluorescein BDA tracer, see Figure 2C), using an Olympus DP-11 system microscope with a x4 and x10 objective lens. We also used dark field microscopy to visualize the anterograde labeling of the BDA. The brightfield and darkfield microscopy of the BDA revealed a very similar pattern of labeling, however the brightfield images produced the clearest and most localized distribution of label. Therefore, the bright field photomicrographs were used for the analysis of anterograde labeling.

#### Microscopic analysis

Initially, the entire forebrain was examined for labeling. Areas of temporal and sensory-motor cortex were found to contain the strongest and most consistent labeling of connections; therefore a more detailed analysis was carried out on these regions to examine the organization of connections. Some results of the sensory-motor cortex analysis were reported previously (Bedwell et al., 2014b) and none of the sensory-motor cortex connections are reported in the present paper.

#### Statistical analysis of the arrangement of connections between PFC and temporal cortex

**Figure 3:**
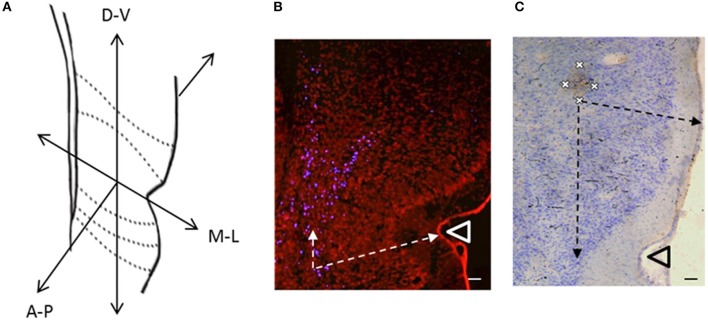
**(A)** Coronal cross section of temporal cortex at −3.3 mm posterior to bregma, depicting the three dimensions in which the locations of labeled cells were recorded (A-P, Anterior-Posterior; M-L, Medial-Lateral; D-V, Dorsal-Ventral). **(B)** Retrogradely labeled cells (blue cells) in temporal cortex produced by an injection of Fluoro-Gold (100 nl) into VO (R6). Arrows indicate measurements of labeled cell locations in the medial-lateral dimension (distance to lateral cortical surface) and dorsal-ventral dimension (distance from rhinal sulcus). **(C)** Anterogradely labeled area (Brown) in temporal cortex produced by an injection of BDA (100 nl) into VO (R7). Crosses indicate the four points on the perimeter at which measurements were taken. Arrows indicate measurements of labeled area location (axon terminals) in the medial-lateral dimension (distance to lateral cortical surface) and dorsal-ventral dimension (distance from rhinal sulcus). Scale bars = 100 μm. Triangles indicate the location of the rhinal sulcus.

We implemented a statistical analysis to determine whether connections between PFC and temporal cortex displayed an ordered arrangement. ImageJ (Wayne Rasband, NIH) was used to determine numerical values representing the location of retrogradely labeled cells in temporal cortex. The dorsoventral and medial-lateral distance (i.e., laminar location) of each retrogradely labeled (Fluoro-Gold) cell was measured from the rhinal sulcus and cortical surface respectively. The anterior-posterior location of each retrogradely labeled cell was also recorded, in terms of distance (mm) from Bregma according to a stereotaxic atlas (Paxinos and Watson, 1998). A similar acquisition was implemented for the anterograde data (DAB visualized BDA projections). Anterogradely labeled areas were defined by intense labeling where a clearly visible area of DAB staining could be seen that covered a grouping of cortical cells (see marked areas in Figures 2C, 3C), isolated DAB labeling in proximity with individual cells was not included (see lower portion of Figure 2C; See Figure S3 of the Supplementary Material for examples of cortical sections where BDA labeling was weak or absent). Four data points were recorded from quadrants at the perimeter of each anterogradely labeled area, denoting its distance (mm) from the rhinal sulcus in dorsoventral and medial-lateral directions (see Figure 3). The anterior-posterior location of each labeled area was also recorded. For the analysis of the location of Fluoro-Ruby axon terminals, which were typically located around the profile of neuronal cell soma, we identified this specific location in terms of x, y, and z coordinates as described above (i.e., expressed as the average location of the neuron).

Labeled cells were grouped according to injection site location. The positional data was found to be normally distributed. Therefore, these data sets were analyzed in SPSS by way of a factorial ANOVA, in order to establish the existence of an effect of injection location on positioning of labeled cells in anterior-posterior, dorsoventral, and medial-lateral dimensions. The relationship between anterograde and retrograde label locations was examined by means of a Two Factor ANOVA. This allowed us to establish the difference between input and output connectivity patterns. All statistical tests were applied with a significance level of 0.05 and confidence intervals of 95%. Within temporal cortex the statistical analysis was applied to (1) 2443 Fluoro-Gold labeled cells arising from 12 rats: PL(*n* = 3), VO(*n* = 3), VLO(*n* = 3), DLO(*n* = 3); (2) 56 BDA labeled points arising from 8 rats: PL(*n* = 2), VO(*n* = 2), VLO(*n* = 2), DLO(*n* = 2) and (3) 733 Fluoro-Ruby labeled points arising from 8 rats: PL(*n* = 2), VO(*n* = 2), VLO(*n* = 2), DLO(*n* = 2). See Table 1 of the Supplementary Material for summary of animals included in the analysis.

## Results

Injections of retrograde and anterograde tracers were made into identical sites within a coronal section of the rat PFC at distance of 4.20 mm anterior to bregma according to Paxinos and Watson (1998) (see Figure 1). The injections were made in a line which followed the curvature of the cortical surface and were largely non-overlapping in terms of spread along the medial-lateral axis (i.e., in terms of injections into orbital cortex), however the dual nature of the injections made the spread larger than would have been expected from the use of single tracers. There was some significant overlap of spread between the injections placed at A and B (albeit in different animals) due to the oval nature of the spread of tracer injection. Using light/fluorescent microscopy, patterns of labeling throughout the temporal cortex region were visualized following the injections of anterograde and retrograde tracer into the PFC cortex (PL, VO, VLO, and DLO—see sites in Figures 1, 2). Following the injections of tracer into the PFC, the anterograde and retrograde labeling observed was largely confined to the ipsilateral hemisphere, thus only the ipsilateral connections are reported.

### Retrograde labeling in the temporal cortex region

The retrograde tracer Fluoro-Gold labeled neuronal cell bodies in the temporal cortex (see Figures 2A,B). Retrograde labeling was seen in regions adjacent to the rhinal sulcus, namely the perirhinal cortex (areas 35 and 36), entorhinal cortex, area Te, and piriform cortex (all areas were defined according to Burwell (2001).

### Anterograde labeling in the temporal cortex region

Biotinylated dextran amines were used to label the axon terminals in the temporal cortex region and is shown by the area visualized by the brown coloration of the DAB-staining (see Figure 2C). Anterograde labeling was seen in the same regions of the temporal cortex as the retrograde labeling, namely the perirhinal cortex (areas 35 and 36), entorhinal cortex and area Te (all areas were defined according to Burwell (2001). The anterograde tracer Fluoro-Ruby was also employed to visualize axon terminals. Fluoro-Ruby was injected at four injection sites, both in terms of single injections and also via dual-injections with Fluoro-Gold (see Table 1 of the Supplementary Material). The pattern of labeling observed was slightly more diffuse than that for BDA Fluorescein but followed the same pattern in terms of the distribution of the label produced.

### The position of anterograde and retrograde labels within the temporal cortex region: location, ordering, and reciprocity

#### Reciprocity

**Figure 4:**
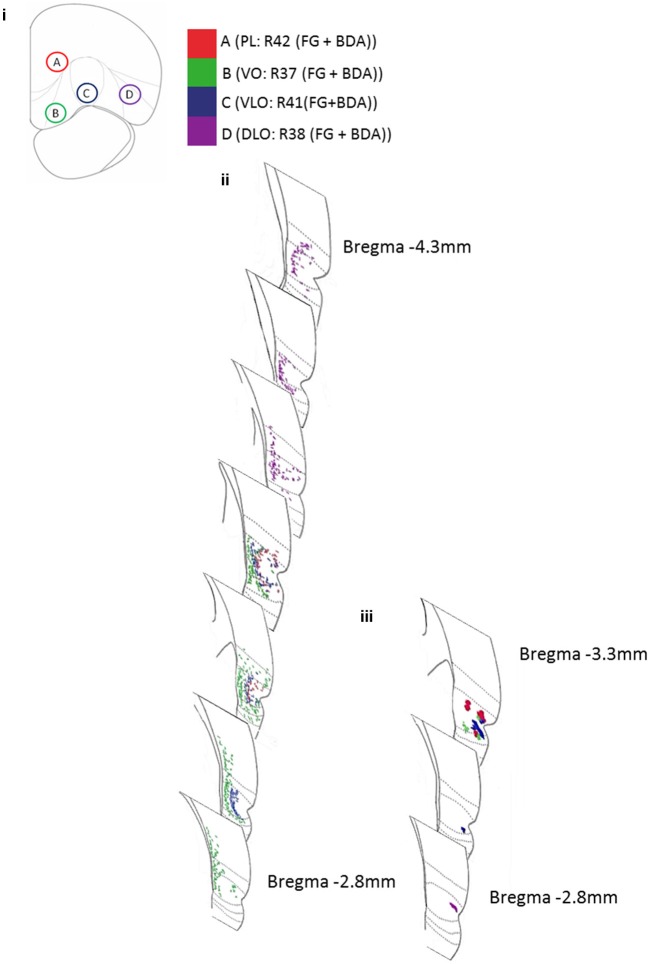
**Diagram representing the amalgamated dual-injection sites of Fluoro-Gold and BDA, and the projection sites in temporal cortex for both retrograde (Fluoro-Gold) and anterograde (BDA) tracer injections from 4 rats**. **(i)** The locations of injection sites A (PL: R42), B (VO: R37), C (VLO:R41), and D (DLO:R38) in PFC. **(ii)** The locations of retrogradely labeled cells in temporal cortex, resultant from 100 nl Fluoro-Gold injections into PFC sites A–D (R37, R38, R41, R42). **(iii)** The locations of anterogradely labeled areas in temporal cortex, resultant from 100 nl BDA (Fluorescein) injections into PFC sites A-D (R37, R38, R41, R42). Borders of entorhinal cortex, 35v, 35d, 36v, 36d, Te are shown (moving dorsally).

The position of anterograde and retrograde labels arising from identical dual-injection sites usually occupied the same cortical region (i.e., entorhinal cortex, perirhinal cortex, piriform, or area Te). Therefore, the connections were largely reciprocal in nature. Retrograde labeling from the injection into PL was found in the same area of PRh as anterograde labeling from the same injection site, indicating reciprocal connections here. Similarly, retrograde labeling produced from the tracer injection into VLO was found in the same region of PRh (35d and 36v) as anterograde labeling from the same injection site, again indicating a reciprocal connection. However, retrograde labeling from the same injection site was not found in the region of PRh (36v) where anterograde labeling from a tracer injection into DLO was found. Retrograde labeling from the tracer injection into DLO was found in regions of PRh (35d, 36v, 36d), but considerably far from the equivalent anterograde labeled area (Figure 4).

#### Location and ordering of PFC inputs and outputs: an analysis of the fine-scale reciprocity of connections

**Figure 5:**
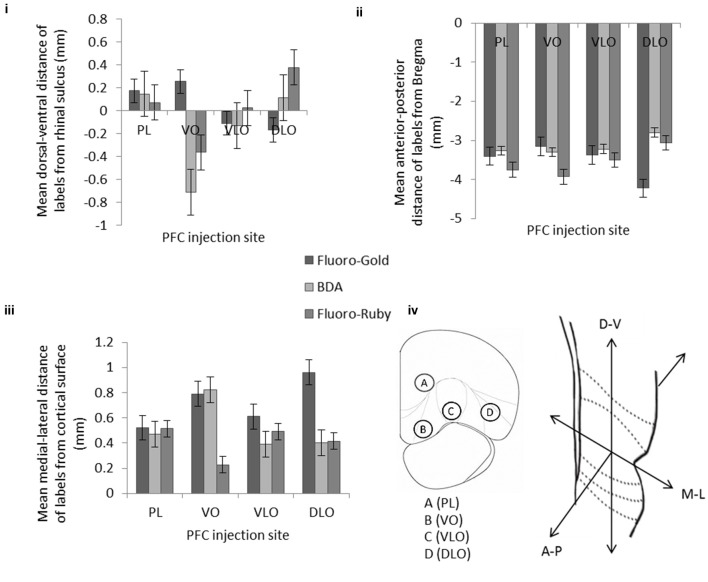
**The effect of injection site on the mean location of retrogradely labeled cells (Fluoro-Gold) and anterograde labeling (BDA and Fluoro-Ruby) within temporal cortex in three orientations: (i) dorsal-ventral, (ii) anterior-posterior, and (iii) medial-lateral**. Error bars = standard error of the mean. **(iv)** Coronal cross section of PFC showing the position of 4 injection sites; PL (A), VO (B), VLO (C), and DLO (D). Coronal cross section of temporal cortex showing the three dimensions in which the locations of labels were recorded. Note that the anterograde labeling (i.e., BDA compared to Fluoro-Ruby) differs, notably in the medial-lateral axis case of VO, but follows the same overall pattern in the other orientations. The retrograde labeling follows a different pattern of labeling (compared to the anterograde labels) in all three orientations. Data includes all of injections detailed in Table 1 of the Supplementary Material (i.e., from both single and dual 100 nl injections). A statistical analysis of this data set is described in the Results Section. **Table 1** of the article contains a breakdown of the mean location of labeling in the constituent *single* and *dual* injection experiments.

To examine the location and ordering of PFC inputs and outputs we needed to analyse the location of retrograde and anterograde label. To do this the location of labeled areas/cells within the temporal cortex was analyzed by first describing in terms of 3D position (in the x, y, and z axes—see Figure 5) and then by analyzing the locations associated with each injection site statistically.

Figure 4 shows the location of anterograde and retrograde labeling in the temporal cortex region following the different injections into the PFC region (data shown from 4 animals—following dual injections of Fluoro-Gold and BDA-Fluorescein). The figure reveals that as injection site was moved laterally from sites B-D the retrograde label occurred increasingly posteriorly within the temporal cortex region. The figure also shows that the retrograde tracer is much broader in its distribution within temporal cortex than the anterograde tracer. This indicates that there is considerable convergence in the feedback circuit projecting from temporal cortex to PFC and returning back to temporal cortex.

The results of the analysis of the location of retrograde and anterograde labels are shown in detail in Figure 5. Figure 5 shows the results for the locations of label following (1) single and dual injections of Fluoro-Gold and BDA and (2) the single and dual injections of Fluoro-Gold and Fluoro-Ruby (the data and animals from this figure are detailed in Table 1 of the Supplementary Material). The location of retrograde and anterograde tracer is shown in 3 axes of orientation (dorsal-ventral, anterior-posterior and medial-lateral). In the graphs the mean location of the label is shown by the bar for each axis, the error bars reveal the SEM. The locations of the anterograde Fluoro-Ruby and BDA labeling appeared similar (see Figures 5i–iii) however a statistical analysis shows that there are differences. A Two Factor ANOVA [injection type(anterograde, retrograde), injection location (A,B,C,D)] showed that there is a significant interaction between BDA and Fluoro-Ruby labeling in 2 axes of orientation [anterior-posterior: *F*_(3, 781)_ = 3.240 *p* = 0.02; medial-lateral: *F*_(3, 781)_ = 14.057 *p* < 0.001]. This indicates that the BDA and Fluoro-Ruby labeling is not identical. However, there is a strong similarity between the mean location and ordering of BDA and Fluoro-Ruby labels in all three orientations (Figures 5i–iii).

We also analyzed how the positioning of Fluoro-Gold labeling compared to (1) BDA labeling and (2) Fluoro-Ruby labeling:

The dorsal-ventral graph (Figure 5i) shows prominent and significant ordering of the Fluoro-Gold (*p* < 0.001), BDA (*p* < 0.001) and Fluoro-Ruby (*p* < 0.001) through sites B–D (VO-DLO) [ANOVA injection, location (A,B,C,D)] – a complete outline of the statistical analysis for this study is included in the complementary material section. This panel also shows that the ordering of Fluoro-Gold (moving ventrally across the graph) differs from that of BDA and Fluoro-Ruby [moving dorsally across the graph, excepting PL(A)]. This differential ordering {Two Factor ANOVA [injection type(anterograde, retrograde), injection location (A,B,C,D)]} is present in the comparisons between (1) Fluoro-Gold and BDA (*p* < 0.001) and (2) Fluoro-Gold and Fluoro-Ruby (*p* < 0.001).

The anterior-posterior graph (Figure 5ii) also shows prominent and significant ordering of the Fluoro-Gold (*p* < 0.001), BDA (*p* < 0.001) and Fluoro-Ruby (*p* < 0.001) through sites B-D (VO-DLO) [ANOVA (injection location (A,B,C,D)]. This panel also shows that the ordering of Fluoro-Gold (moving posteriorly across the graph) differs from that of BDA and Fluoro-Ruby (moving anteriorly across the graph, again excepting PL(A)). This differential ordering {Two Factor ANOVA [injection type (anterograde, retrograde), injection location (A,B,C,D)]} is present in the comparisons between (1) Fluoro-Gold and BDA (*p* < 0.001) and (2) Fluoro-Gold and Fluoro-Ruby (*p* < 0.001).

We also found evidence for differential ordering of inputs and outputs in the medial-lateral (i.e., laminar) axis of orientation; this is entirely consistent with the idea that cortical inputs and outputs arise from different cortical layers (Barbas, 1995). Finally it is clear from viewing Figures 4, 5 that although the corresponding anterograde and retrograde tracers do occupy the same approximate cortical regions they do not in general occupy the same intra-areal sites. Table 1 (article) provides a breakdown of the mean locations of retrograde and anterograde labeling in the temporal cortex following *single* and *dual* injections of Fluoro-Gold, BDA and Fluoro-Ruby into the PFC divisions (this is data that was amalgamated in Figure 5).

**Table 1:** **The mean location of labeling in temporal cortex, in three axes of orientation following** ***single*** **and** ***dual*** **tracer injections of Fluoro-Gold, Fluoro-Ruby, and BDA made into PL, VO, VLO, and DLO in prefrontal cortex**.

		**PL (A)**		**VO (B)**		**VLO (C)**		**DLO (D)**	
		**Mean**	**SEM**	**Mean**	**SEM**	**Mean**	**SEM**	**Mean**	**SEM**
Dorsal-Ventral	Fluoro-Gold Single Injection	0.14	0.03	0.37	0.02	−0.16	0.05	−0.17	0.03
	Fluoro-Gold Dual Injection (with BDA)	0.18	0.04	0.4	0.14	−0.16	0.04	−0.17	0.07
	Fluoro-Gold Dual Injection (with Fluoro-Ruby)	0.22	0.02	0.33	0.03	−0.04	0.03	−0.16	0.11
	BDA Single Injection	0.22	0.01	−0.21	0.01	−0.06	0.01	0.01	0
	BDA Dual Injection	0.12	0.14	−0.88	0.29	−0.15	0.12	0.21	0.04
	Fluoro-Ruby Single Injection	−0.07	0.05	−0.5	0.14	−0.01	0.05	0.48	0.12
	Fluoro-Ruby Dual Injection	0.16	0.04	−0.09	0.04	0.077	0.05	0.18	0.13
Anterior-Posterior	Fluoro-Gold Single Injection	−3.31	0.02	−3.1	0.01	−3.4	0.02	−4.35	0
	Fluoro-Gold Dual Injection (with BDA)	−3.38	0.02	−3.23	0.02	−3.38	0.02	−4.1	0.02
	Fluoro-Gold Dual Injection (with Fluoro-Ruby)	−3.53	0.03	−3.25	0.01	−3.34	0.02	−3.96	0.02
	BDA Single Injection	−3.14	0	−3.3	0	−3.14	0	−2.8	0
	BDA Dual Injection	−3.3	0	−3.3	0	−3.25	0	−2.8	0
	Fluoro-Ruby Single Injection	−4.41	0.01	−4.21	0.01	−3.6	0.03	−3.1	0.01
	Fluoro-Ruby Dual Injection	−3.3	0	−3.36	0.02	−3.33	0.03	−3	0.02
Medial-Lateral	Fluoro-Gold Single Injection	0.57	0.01	0.66	0.01	0.67	0.02	0.7	0.01
	Fluoro-Gold Dual Injection (with BDA)	0.44	0.01	1.6	0.04	0.56	0.02	0.88	0.06
	Fluoro-Gold Dual Injection (with Fluoro-Ruby)	0.48	0.01	0.65	0.02	0.59	0.02	1.74	0.05
	BDA Single Injection	0.52	0.02	0.5	0.03	0.43	0.03	0.53	0.09
	BDA Dual Injection	0.45	0.04	0.93	0.1	0.38	0.03	0.28	0.06
	Fluoro-Ruby Single Injection	0.5	0.03	0.13	0.01	0.52	0.03	0.01	0
	Fluoro-Ruby Dual Injection	0.52	0.02	0.43	0.03	0.45	0.03	1.21	0.08

The table features the same data as that shown in Figure 5 but also includes the individual experiments (e.g., in the case of Fluoro-Gold: the results from the single and both dual injection experiments are tabulated here). Note the general agreement between the BDA and Fluoro-Ruby locations (except in the medio-lateral orientation in the case of VO). There are several individual differences between the mean locations of Fluoro-Gold and the anterograde labeling (see the dorso-ventral orientation—VO and the anterior-posterior orientation—DLO). SEM = standard error of the mean.

To conclude, the results show that the PFC input connections are clearly ordered in the anterior-posterior axis of the temporal cortex. The ordering seen is clear between injections sites B-D, i.e., between the VO and DLO subdivisions of the PFC. Similarly the PFC output connections to the temporal cortex also displayed clear ordering between VO and DLO in the dorsal-ventral axis. Taken together the results show that there is a generalized reciprocity in terms of PFC inputs and outputs, i.e., that the same cortical regions send and receive projections from the PFC. However, when viewed at a finer resolution we observed that the cortical inputs and outputs to the PFC display different patterns of ordering.

## Discussion

In summary, we found an ordered arrangement of input and output connections in the PFC. We also found evidence for a differential ordering of input and output connections, indicating that specific PFC inputs and outputs are connected to different columnar regions of temporal cortex.

### Methodological considerations

In this study it should be noted that due to the relatively large volume of tracer injectate and use of pressure injections we cannot entirely exclude the possibility of some uptake of tracers from damaged fibers of passage. The present results are based on dual and single injection of tracers into the same injection site. The dual injection approach is superior to that of single injection studies, however there is always the possibility that different tracers may be subject to different levels of injection spread and uptake. Injection spread can be relatively easily measured (the spread of our retrograde tracer was larger than that of our anterograde BDA or Fluoro-Ruby tracers) but uptake is more difficult to quantify. A key feature of our results is that, although the location and distribution spread of our projections differed (possibly due to the differences outlined above), they were also centered upon different columnar regions of cortex. It is this key feature (i.e., the different centers of the projection sites) that points to non-reciprocity of fine-scale PFC connections.

### Anterograde and retrograde labeling following dual injections into PFC

Following dual injections of anterograde and retrograde tracer into PFC sites we found significant labeling of anterograde and retrograde connections in temporal cortex. We observed anterograde label located primarily within the perirhinal cortex. Retrograde label was found to be more widespread: within area Te, perirhinal cortex, entorhinal cortex, and piriform cortex. Previous studies have also reported prominent projections from temporal cortex to PFC and in the return projection.

### Ordering of connections within the cerebral cortex

Our findings show that the projections from PFC to temporal cortex and from temporal cortex to PFC are ordered. However, the ordering seen is different for the input and output connections. In a previous study of the PFC projections to temporal cortex the authors reported topographical ordering (Kondo and Witter, 2014). The labeling of anterograde projections that we found was largely confined to the perirhinal cortex region and this is consistent with this previous report by Kondo and Witter. We did not observe a very clear ordering of PFC projections to the perirhinal cortex region but did observe that anterograde label occurred more dorsally in the perirhinal region as PFC injection site traversed from medial-lateral: VO, VLO, DLO. This finding has been reported in the projections from the perirhinal region to MO and VO (in this experiment the retrograde tracer Fluoro-Gold was used) (Hoover and Vertes, 2011). The report by Kondo and colleagues does not present the same ordering, however we observed similar patterns of labeling to those reported by Kondo and colleagues. Our strongest ordering appeared in the return projections from temporal cortex to PFC (visualized with retrograde labeling).

Previous studies have also reported ordering in the connections from PFC to the striatum (Sesack et al., 1989; Berendse et al., 1992), the thalamus (Groenewegen, 1988) and in the connections between PFC regions (Conde et al., 1995). At a broader level previous studies have reported the topological ordering of rodent PFC to a number of connected cortical and subcortical sites (Sesack et al., 1989; Olsen and Musil, 1992). Topographic ordering of connections is also reported in the connections between PFC and pyriform cortex (Datiche and Cattarelli, 1996). Taken together, there is widespread evidence that PFC connections display clear ordering with both cortical and subcortical sites. This is consistent with other regions of the cerebral cortex where topographically/topologically arranged connections (Henry and Catania, 2006; Thivierge and Marcus, 2007; Aronoff et al., 2010) are hypothesized to support physiological maps (Woolsey, 1967; Welker, 1971; Hafting et al., 2005; Marshel et al., 2011; Dias et al., 2014). To our knowledge this study is the first to simultaneously report on the ordering of anterograde and retrograde labeling in temporal cortex following PFC injections.

### Reciprocity of PFC input and output connections to temporal cortex

We observed reciprocal connectivity in the pathway between PFC and temporal cortex; anterograde and retrograde label were found in overlapping regions of perirhinal cortex following dual tracer injections into VO and VLO. However, retrograde injections consistently labeled additional regions to their anterograde counterparts. This was most apparent with the labeling from injections into DLO, where anterogradely labeled output connections did not appear in the same cytoarchitectural regions as retrogradely labeled input connections from the same injection site. The labeling displayed areas of reciprocity, but also shows a large number of retrograde labels in temporal cortex that did not have reciprocal anterograde connections. Reciprocity is known to occur widely across the cerebral cortex (Felleman and Van Essen, 1991) and has been reported to occur in temporal cortex connections of rodent cerebral cortex (Agster and Burwell, 2009, 2013).

### Fine-scale reciprocity and the differential ordering of input and output connections

The differential ordering of connections described here indicates that retrograde and anterograde labels (and subsequent PFC inputs and outputs) occur in different columnar regions of temporal cortex. This can be compared to similar studies in sensory, motor and association cortex.

In sensory and motor cortex there is strong evidence for both highly ordered reciprocal connections and for weakly reciprocal or non-reciprocal connections. The connections between primary somatosensory cortex (S1) and secondary somatosensory cortex (S2) (Burton and Kopf, 1984; Henry and Catania, 2006), S1 and primary motor cortex (M1) (Porter and White, 1983; Aronoff et al., 2010) and between S2 and M1 (Porter and White, 1983; Burton and Kopf, 1984) are reported to be highly reciprocal in several mammalian species. To qualify this further, the same columnar cortical projection sites contain both input and output connections to the specified target site.

However, if we consider the situation of connections between sensory-motor cortex and other brain sites there is evidence for weaker reciprocal connectivity. In one study, the projection from motor cortex to the ipsilateral striatum did not display a return projection (Porter and White, 1983). Another study involving injections of anterograde tracer or retrograde tracer into PFC subdivisions showed that labeling often occurred in distinct columnar regions of sensory-motor cortex (Bedwell et al., 2014b).

The connections of temporal association cortex appear to show a similar profile (to that of sensory-motor cortex) with some connections displaying tight, parallel-organized input and output connections and others displaying weakly reciprocal or non-reciprocal connections. The connections between pyriform and perirhinal cortex, between lateral entorhinal and perirhinal cortex (Canto et al., 2008) and between pyriform cortex and PFC are reported to be strongly reciprocal (Datiche and Cattarelli, 1996; Agster and Burwell, 2009). The connections between lateral entorhinal cortex and secondary motor cortex (Agster and Burwell, 2009) and some connections between PFC and temporal cortex (Kealy and Commins, 2011) are not reported to be reciprocal at all. A problem with the interpretation of these results is that most studies have not sought to identify the fine-scale reciprocity of cortical inputs and outputs, therefore it is difficult to conclude whether parallel-arranged input and output connections are a feature of specific cortical connections and pathways.

It is noteworthy that in the present study the connections between prelimbic cortex and temporal cortex appeared to be largely reciprocal (see Figure 4), unlike the other PFC regions under investigation. A previous study has reported that mPFC connections to PFC regions immediately anterior or posterior are highly reciprocal (Conde et al., 1995). Further, our previous study of PFC connections to the sensory-motor region agreed with this finding, reporting that the connections between prelimbic and secondary motor cortex were reciprocal (Bedwell et al., 2014a). Taken together, these findings suggest that dorsal mPFC displays reciprocal connections between local and long-range cortical sites. By contrast, the orbital PFC shows differential ordering of connections and therefore much weaker reciprocal connections between PFC and sensory-motor (Bedwell et al., 2014b) and temporal cortex connections (present report). The significance of this difference within PFC is not currently clear.

### Relevance of the results to the subdivisions of temporal cortex

Temporal Cortex has been previously defined according to different subdivisions, such as Te1 (primary auditory cortex), Te2, and Te3 (Arnault and Roger, 1990; Shi and Cassell, 1999). Our tracer injections into VO and DLO produced retrograde labeling in Te3. We found no BDA labeling in Te3 from the same PFC injection sites, indicating non-reciprocal connections from Te3 to VO and DLO. None of our tracer injections produced labeling in the other temporal regions: Te1 and Te2. Our finding of non-reciprocal connections between PFC and Te3 adds to the previous findings of non-reciprocal labeling in the brainstem and sub-cortical areas, following tracer injections into Te3 and Te2 (Arnault and Roger, 1990). Arnault and Roger (1990) also reported that Te2 and Te3 have dense reciprocal connections with the medial geniculate complex. There have also been reports of reciprocal connections from ventral Te (Te2 and Te3) to the dorsal aspect of perirhinal cortex (Shi and Cassell, 1999). The same study identified broadly reciprocal connections from perirhinal cortex to multiple cortical regions, these findings are similar to the broad scale reciprocity we have identified between PFC and temporal cortex. Topographic ordering has been described in the connections between Te1 and the medial geniculate complex (Arnault and Roger, 1990). A clear ordering of Te3 labeling cannot be seen from our findings, Arnault and Roger (1990) similarly described no precise topographic ordering of Te2 and Te3 connections to the medial geniculate complex.

### Significance of non-reciprocity for behavior

Studies of the connections between PFC and the thalamus in primates have described non-reciprocal connections and linked this to feed-forward loops associated with PFC (McFarland and Haber, 2002). It is possible that feed-forward loops could be implicated in the memory functions associated with PFC in a variety of species (Fuster, 2001; Barker and Warburton, 2008).

In conclusion, we found evidence for highly organized topological connections from PFC to temporal connections and in return projections from temporal cortex to PFC. However, the ordering of the input and output connections frequently did not match and reflected a differential ordering of anterograde and retrograde label. In addition we found that following injections into the PFC, anterograde and retrograde labels were not localized within the same columnar regions indicating a mismatch between the organization of PFC inputs and outputs. The results of this study highlight the need for further investigation into precise ordering, alignment and fine-scale reciprocity of association cortex connections in rodents and higher mammalian species.

### Conflict of interest statement

The authors declare that the research was conducted in the absence of any commercial or financial relationships that could be construed as a potential conflict of interest.
