# Supplementary material for: The topology of connections between rat prefrontal and temporal cortices
Source: Front Syst Neurosci. 2015 May 20;9:80. doi: 10.3389/fnsys.2015.00080 (PMC4438597; doi:10.3389/fnsys.2015.00080)
Supplement: Supplementary file 1 [file DataSheet1.DOCX]

Supplementary Material

Results Section: Statistical Analysis

The following sections detail the statistical evidence for the ordering and alignment of PFC inputs and output connections occurring across 3 axes of orientation of the temporal cortex region.

*Statistical Evidence for the Differential Ordering of Input and Output Connections*

A factorial ANOVA was applied to the locations of retrogradely labelled cells in each axis of orientation. This was repeated for the locations of anterogradely labelled areas and axon terminals. A 2 factor ANOVA (injection type[anterograde, retrograde], injection location [PL(A),VO(B),VLO(C),DLO(D)]) was applied to the locations in order to establish the relationship between input and output connections.

The statistical analyses were applied to two datasets, Fluoro-Gold compared to BDA labelling and Fluoro-Gold compared to Fluoro-Ruby labelling. All analyses contained data from dual and single tracer injections combined.

**Analysis of Location and Ordering in the Anterior-Posterior Axis:**

**For the comparison of Fluoro-Gold and BDA:** The factorial ANOVA revealed a significant main effect of injection site on the anterior-posterior distance from bregma of both anterograde (BDA) (*F*_(3,52)_=145.299 *p*<.001) and retrograde (Fluoro-Gold) (F_(3,2439)_=2546.788 p<.001) labelling in temporal cortex. The two factor ANOVA revealed a significant interaction effect between input and output connections (*F*_(3,2472)_=82.099 *p*<.001).

**For the comparison of Fluoro-Gold and Fluoro-Ruby:** A factorial ANOVA revealed a significant main effect of of injection site on the anterior-posterior distance from bregma of both anterograde (Fluoro-Ruby) (*F*_(3,729)_=208.706 *p*<.001) and retrograde (Fluoro-Gold) (F_(3,2439)_=2546.788 p<.001) labelling in temporal cortex. The two factor ANOVA revealed a significant interaction effect between input and output connections (*F*_(3,3149)_=1445.157 *p*<.001). The significant effect of injection site indicates a difference in the location of the labelling of tracers according to injection site, demonstrating an ordered arrangement (see Fig.5). The significant interaction between input and output connections shows that the anterograde and retrograde labelling varies with respect to each other and indicates differential ordering. This analysis shows significant ordering of the retrograde label and anterograde label within the temporal cortex.

**Analysis of Location and Ordering in the Dorsal-Ventral Axis:**

**For the comparison of Fluoro-Gold and BDA:** The factorial ANOVA revealed a significant main effect of injection site on the dorsoventral distance from the rhinal sulcus of both anterograde (BDA) (*F*_(3,52)_=7.126 *p*<.001) and retrograde (Fluoro-Gold) (F_(3,2439)_=93.384 p<.001)  labelling in the temporal cortex. The 2 factor ANOVA revealed a significant interaction effect between input and output connections (*F*_(3,2472)_=8.973 *p*<.001).

**For the comparison of Fluoro-Gold and Fluoro-Ruby:** The factorial ANOVA revealed a significant main effect of injection site on the dorsoventral distance from the rhinal sulcus of both anterograde (Fluoro-Ruby) (*F*_(3,729)_=208.706 *p*<.001) and retrograde (Fluoro-Gold) (F_(3,2439)_=93.384 p<.001)  labelling in the temporal cortex. The 2 factor ANOVA revealed a significant interaction effect between input and output connections (*F*_(3,3149)_=63.350 *p*<.001). The significant effect of injection site indicates a difference in the location of the labelling of tracers according to injection site, demonstrating an ordered arrangement (see Fig.5). The significant interaction between input and output connections shows that the anterograde and retrograde labelling vary with respect to each other and indicates differential ordering. This analysis shows significant ordering of the anterograde and retrograde label within temporal cortex and the significant interaction indicates differential ordering of the anterograde and retrograde labels in this axis.

**Analysis of Location and Ordering in the Medial-Lateral Axis:**

**For the comparison of Fluoro-Gold and BDA:** The factorial ANOVA revealed no significant main effect of injection site on the medial-lateral distance from the cortical surface of both anterograde (BDA) (*F*_(3,52)_=1.393 *p*=.255) and a significant main effect of retrograde (Fluoro-Gold) (F_(3,2439)_=122.898 p<.001)  labelling in the temporal cortex. The 2 factor ANOVA showed a significant interaction effect between input and output connections (*F*_(3,2472)_=26.626 *p*<.001).

**For the comparison of Fluoro-Gold and Fluoro-Ruby:** The factorial ANOVA revealed a significant main effect of injection site on the medial-lateral distance from the cortical surface of both anterograde (Fluoro-Ruby) (*F*_(3,729)_=18.158 *p*<.001) and retrograde (Fluoro-Gold) (F_(3,2439)_=122.898 p<.001)  labelling in the temporal cortex. The 2 factor ANOVA showed a significant interaction effect between input and output connections (*F*_(3,3149)_=67.503 *p*<.001). The significant effect of injection site indicates a difference in the location of the labelling according to the injection site (see Fig.5). The significant interaction between input and output connections indicates that the input and output connections vary with respect to one another. This analysis shows significant ordering of retrograde and anterograde labelling within the temporal cortex in this axis of orientation.

Following the 2-factor analysis outlined above we performed t-tests for independent samples on the positions of (1) the Fluoro-Gold labelling compared to the BDA labelling and (2) the Fluoro-Gold labelling compared to the Fluoro-Ruby labelling (the same data sets analysed by the 2 factor ANOVA were used here aswell). This was performed for each injection location and used an independent samples approach because single injections compared across different animals were used as well as dual injections. The results of these analyses are reported in table 2 of the supplementary material (see below). They show that the anterograde and retrograde labelling differ following injections into all 4 prefrontal cortex sites. The strongest of these differences occurs following the injections into VO (B) and DLO (D). The analyses following injections into PL (A) and VLO (C) shows that there are fewer significant differences between the retrograde and anterograde labels, however when the 3 orientations are considered collectively significant differences are present.

Complementary Figures to the Manuscript

In the following section there are three additional figures. Figure 1 is additional to figure 1i and ii in the accompanying manuscript and shows the separate labelling for Fluoro-Gold and Fluorescein Biotinylated Dextran Amine (BDA) dual injections.

Figure 2 shows the locations of the dual injections of Fluoro-Gold and Fluoro-Ruby.

Figure 3 shows brain sections with weak DAB labelling of BDA following the injections into the prefrontal cortex. Note that the temporal cortex region featured does not show intense region(s) of brown (i.e. DAB labelling) that cover a grouping of cortical cells. This second figure shows that BDA labelling was not seen across all regions of the temporal cortex.

In addition, the injection sites for the single injections of Fluoro-Gold and Fluorescein BDA are shown in Figure 1iv of Bedwell et al 2014b. The analysis of temporal cortex projections was not reported in this previous paper.


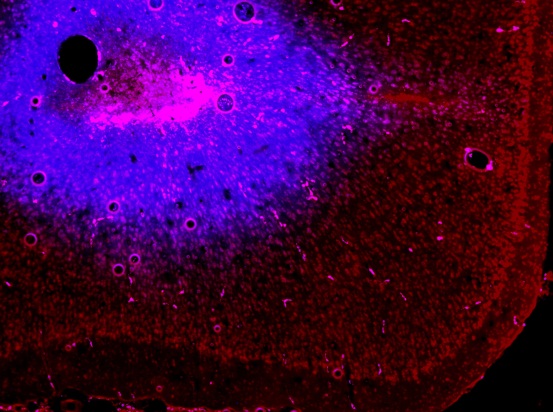


**ii**


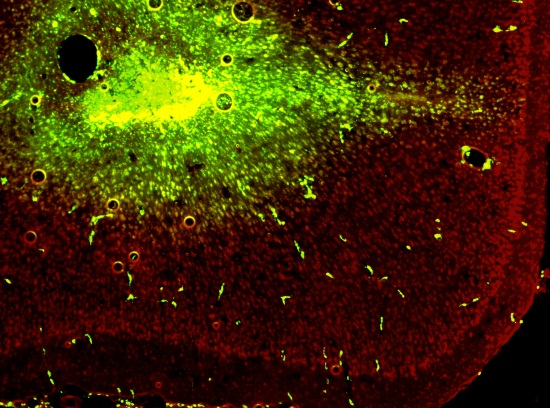


**i**


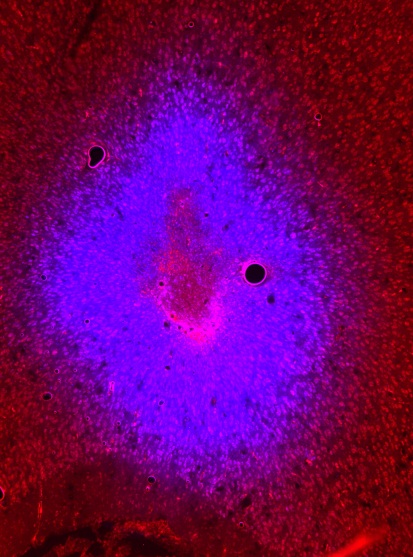


**iv**


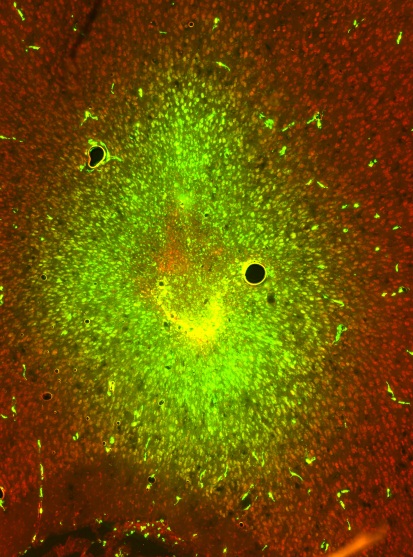


**iii**

Figure 1. (i & ii) Coronal sections of prefrontal cortex showing site of dual-injection of (i) 100nl Fluorescein BDA (green) and (ii) 100nl Fluoro-Gold (blue) into VO (case R37). (iii & iv) Coronal sections of prefrontal cortex showing site of dual-injection of (iii) 100nl Fluorescein BDA (green) and (iv) 100nl Fluoro-Gold (blue) into DLO (case R38). Red = propidium iodide. Scales bars = 200µm.


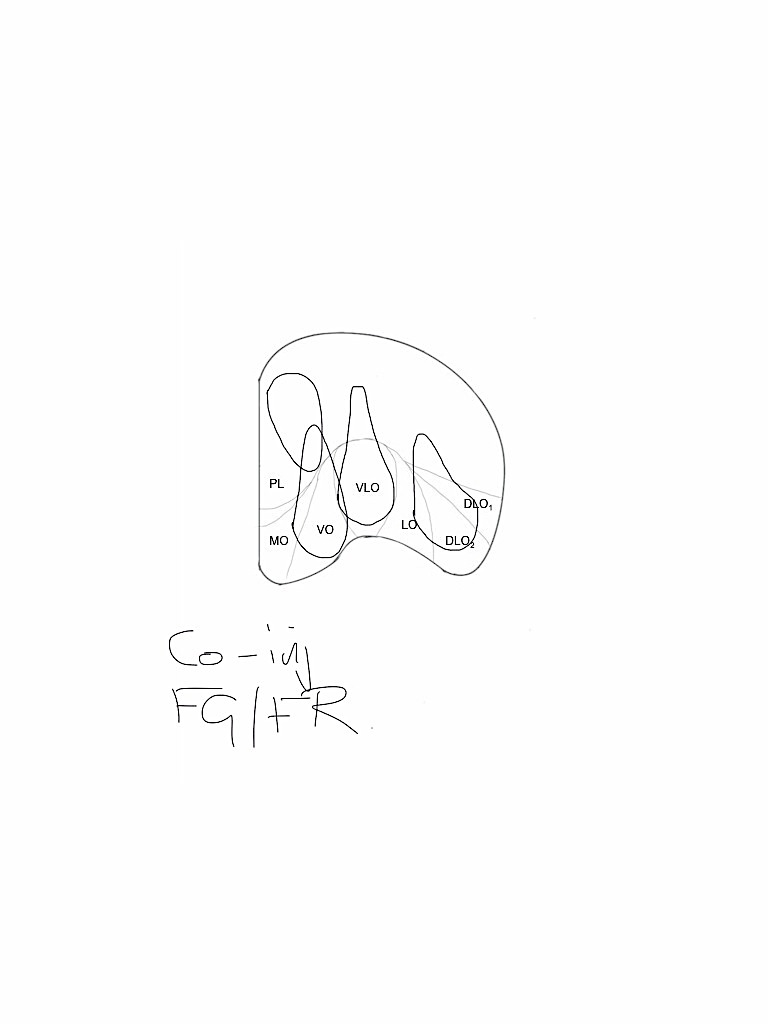


IL

R44

R39

R43

R40

Figure 2. Coronal cross section of PFC showing location and spread of Fluoro-Gold (100nl) and Fluoro-Ruby (100nl) dual-injection sites in PL (injection A:R44), VO (injection B:R39), VLO (injection C:R43) and DLO (injection D:R40) in the left hemisphere.

**ii**

**i**


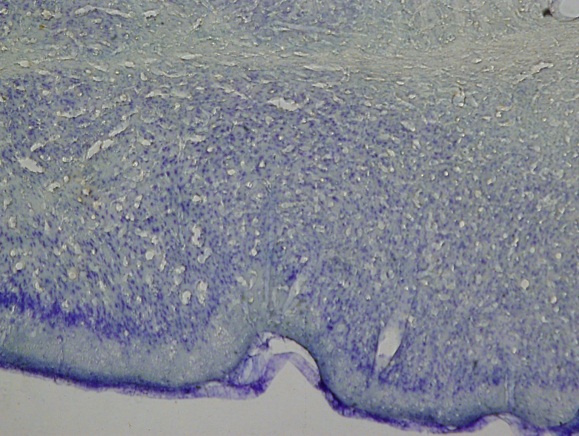

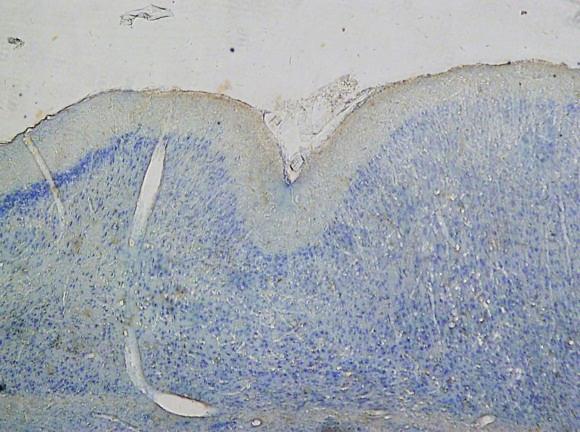


Figure 3. Coronal sections showing areas of temporal cortex where no areas of intense anterograde (BDA) labelling (brown) were found. (i) Thionin (blue) stained temporal cortex section resultant from injection of 100nl BDA into PL (R3). (ii) Thionin (blue) stained temporal cortex section resultant from co- injection of 100nl BDA and 100nl Fluoro-Gold into DLO (R38). Arrows denote the location of the rhinal sulcus. Scale bars=200µm.

| Rat ID | AP | ML | Height | Tracer | volume | Hemisphere(L/R) | Type | Region |
| --- | --- | --- | --- | --- | --- | --- | --- | --- |
| R1 | 3.7 | 2.2 | -3.2 | BDA Fluorescein | 100nl | Right | single | VLO |
| R3 | 3.7 | 1.2 | -2.4 | BDA Fluorescein | 100nl | Right | single | PL |
| R4 | 3.7 | 2.2 | -3.2 | Fluoro-Gold | 100nl | Right | single | VLO |
| R5 | 3.7 | 3.2 | -3.2 | Fluoro-Gold | 100nl | Right | single | DLO |
| R6 | 3.7 | 1.2 | -3.2 | Fluoro-Gold | 100nl | Right | single | VO |
| R7 | 3.7 | 1.2 | -3.2 | BDA Fluorescein | 100nl | Left | single | VO |
| R7 | 3.7 | 1.2 | -2.4 | Fluoro-Gold | 100nl | Right | single | PL |
| R8 | 3.7 | 3.2 | -3.2 | BDA Fluorescein | 100nl | Right | single | DLO |
| R9 | 3.7 | 2.2 | -3.2 | Fluoro-Ruby | 100nl | Right | single | VLO |
| R19 | 3.7 | 3.2 | -3.2 | Fluoro-Ruby | 100nl | Right | single | DLO |
| R20 | 3.7 | 1.2 | -3.2 | Fluoro-Ruby | 100nl | Right | single | VO |
| R29 | 3.7 | 1.2 | -2.4 | Fluoro-Ruby | 100nl | Right | single | PL |
| R37 | 3.7 | 1.2 | -3.2 | Fluoro-Gold | 100nl | Left | dual | VO |
| R37 | 3.7 | 1.2 | -3.2 | BDA Fluorescein | 100nl | Left | dual | VO |
| R38 | 3.7 | 3.2 | -3.2 | Fluoro-Gold | 100nl | Left | dual | DLO |
| R38 | 3.7 | 3.2 | -3.2 | BDA Fluorescein | 100nl | Left | dual | DLO |
| R39 | 3.7 | 1.2 | -3.2 | Fluoro-Gold | 100nl | Left | dual | VO |
| R39 | 3.7 | 1.2 | -3.2 | Fluoro-Ruby | 100nl | Left | dual | VO |
| R40 | 3.7 | 3.2 | -3.2 | Fluoro-Gold | 100nl | Left | dual | DLO |
| R40 | 3.7 | 3.2 | -3.2 | Fluoro-Ruby | 100nl | Left | dual | DLO |
| R41 | 3.7 | 2.2 | -3.2 | Fluoro-Gold | 100nl | Right | dual | VLO |
| R41 | 3.7 | 2.2 | -3.2 | BDA Fluorescein | 100nl | Right | dual | VLO |
| R42 | 3.7 | 1.2 | -2.4 | Fluoro-Gold | 100nl | Left | dual | PL |
| R42 | 3.7 | 1.2 | -2.4 | BDA Fluorescein | 100nl | Left | dual | PL |
| R43 | 3.7 | 2.2 | -3.2 | Fluoro-Gold | 100nl | Left | dual | VLO |
| R43 | 3.7 | 2.2 | -3.2 | Fluoro-Ruby | 100nl | Left | dual | VLO |
| R44 | 3.7 | 1.2 | -2.4 | Fluoro-Gold | 100nl | Left | dual | PL |
| R44 | 3.7 | 1.2 | -2.4 | Fluoro-Ruby | 100nl | Left | dual | PL |

Table 1. Stereotaxic location of tracer injections used in the statistical analyses for each individual rat. Stereotaxic location in terms of anterior-posterior distance with respect to bregma (AP), medial lateral distance with respect to bregma (ML) and height with respect to the cortical surface (all in mm). The tracer type, volume, hemisphere, injection type (single/dual) and target PFC sub-region is also provided. ​

| PFC injection site | Fluoro-Gold cf BDA | | | Fluoro-Gold cf Fluoro-Ruby | | |
| --- | --- | --- | --- | --- | --- | --- |
|  | Dorsal-Ventral | Anterior-Posterior | Medial-Lateral | Dorsal-Ventral | Anterior-Posterior | Medial-Lateral |
| PL (A)  t value  p value | ns | s | ns | s | s | ns |
|  | 0.282 | -6.014 | 1.159 | 2.889 | 7.460 | 0.399 |
|  | 0.778 | <0.001 | 0.247 | 0.004 | <0.001 | 0.690 |
| VO (B)  t value  p value | s | s | ns | s | s | s |
|  | 5.170 | 24.399 | -0.089 | 7.424 | 27.963 | 28.691 |
|  | 0.001 | <0.001 | 0.929 | <0.001 | <0.001 | <0.001 |
| VLO (C)  t value  p value | ns | s | s | s | s | s |
|  | 0.166 | -6.554 | 8.601 | -3.040 | 4.627 | 5.606 |
|  | 0.868 | <0.001 | <0.001 | 0.002 | <0.001 | <0.001 |
| DLO (D)  t value  p value | s | s | s | s | s | s |
|  | -5.371 | -157.192 | 7.729 | -5.722 | -69.597 | 10.121 |
|  | <0.001 | <0.001 | <0.001 | <0.001 | <0.001 | <0.001 |

Table 2. The results independent t-tests between retrograde (Fluoro-Gold) and anterograde (BDA and Fluoro-Ruby) labelling in temporal cortex (in 3 axes of orientation) produced by tracer injections (dual and single injections) into PL, VO, VLO and DLO. s denotes significant, ns denotes not significant.
